# HIV-related Discriminatory Attitudes of Healthcare Workers in Bangladesh

**DOI:** 10.3329/jhpn.v28i2.4892

**Published:** 2010-04

**Authors:** Mohammad Bellal Hossain, Kippax Susan

**Affiliations:** ^1^ Public and Environmental Health Research Unit, Department of Public Health and Policy, London School of Hygiene & Tropical Medicine, 51 Bedford Square, London, WC1B 3DP, UK; ^2^ National Centre in HIV Social Research, Robert Webster Building, University of New South Wales, Sydney, NSW 2052, Australia

**Keywords:** Acquired immunodeficiency syndrome, Cross-sectional studies, Discrimination, Healthcare workers, Human immunodeficiency virus, Stigma, Bangladesh

## Abstract

This study aimed at identifying the level of HIV-related discriminatory attitudes and related factors in a purposively-selected sample of healthcare workers (HCWs) in Bangladesh. In total, 526 HCWs from a number of hospitals and healthcare centres were interviewed using a structured questionnaire. A moderate level of discriminatory attitudes was observed. The factors associated with a high level of such attitudes among the HCWs were: high level of irrational fear about HIV and AIDS; working in teaching hospital rather than in non-teaching hospital and diagnostic centres; low level of education; and being male. The results indicate that programmes to reduce irrational fear about transmission of HIV are urgently needed.

## INTRODUCTION

Discriminatory attitudes towards people living with HIV (PLHIV) among healthcare workers (HCWs) have been observed in many countries (1–8). There has been no systematic study of discriminatory attitudes among HCWs, and to date, the only information available in Bangladesh in this regard is anecdotal evidence and the occasional newspaper reports. As the consequences of discriminatory attitudes are severe in terms of both public health and human rights (8), this study aimed at fulfilling this gap.

Discrimination by HCWs towards PLHIV includes: HIV testing without consent; breaches of confidentiality; denial of treatment and care; refusal of admission to a hospital; refusal to operate or assist in clinical procedures; cessation of ongoing treatment; early discharge from hospital; judgemental attitudes of hospital workers; physical isolation in the ward; restrictions on movement around the ward or room; restricted access to shared facilities; denial of hospice facilities; refusal to lift or touch the dead body of an HIV-positive person; and reluctance to provide transport for the dead body of an HIV-positive person (2, 9, 10).

The concept ‘discrimination’ (action) is often equated with stigma (attitudes). However, the reality is not always like that. Some researchers have argued that discrimination is similar to enacted stigma which refers to the ‘real experience of discrimination’ (11, 12). Major and O'Brien have argued that discrimination is an instrument of stigmatization (13) while Collymore has stated that stigma and discrimination are two separate entities but closely linked (14). This study adopted the the oretical position that discrimination is an outcome of stigmatization (13, 15) and attempted to measure discrimination using hypothetical questions about readiness of HCWs to interact with or provide healthcare services to PLHIV (16). The principal assumption underlying this approach is that refusal to interact or provide treatment is the reflection of discrimination.

## MATERIALS AND METHODS

### Study design and recruitment of participants

The original study from which the findings presented in this paper were taken was designed to identify the levels and correlates of different aspects of stigmatizing and discriminatory attitudes among HCWs and to document the real-life experience of PLHIV. However, in this paper, only the discriminatory attitudes of HCWs are presented.

### Recruitment and procedure

The study was cross-sectional in nature. Five hundred twenty-six HCWs (315 males, 211 females) interviewed for the study were recruited from the three cities (Dhaka, Chittagong, and Sylhet) of Bangladesh from the following different types of healthcare settings: teaching hospitals; non-teaching hospitals; and HIV diagnostic centres. The sample was purposively selected, and all HCWs in the three settings were asked to participate. Trained medical and social science graduates interviewed the HCWs face-to-face. Data were collected during February-May 2005.

### Questionnaire and measures

A structured questionnaire with some open-ended questions was developed for data collection which covered the following: sociodemographic and religious variables; contact with HIV-positive people in the workplace; knowledge about HIV and AIDS; irrational fear about transmission of HIV; and discriminatory attitudes.

### Measures

#### Discriminatory attitudes

The dependent variable—discriminatory attitudes—was measured via 16 items (Table 1) selected covering both social- and healthcare-related discriminatory attitudes towards PLHIV. The items were selected from previous research (1, 3, 17–22). The HCWs were asked to rate each item on a five-point Likert scale, indicating their agreement or disagreement (1=Disagree strongly; 2=Disagree somewhat; 3=Neither agree nor disagree; 4=Agree somewhat; and 5=Strongly agree). The average score on the discriminatory attitudes scale was 36.4, ranging from 16 to 80. The higher the score on this scale, the higher the level of discrimination. The reliability coefficient of this scale was 0.92, indicating high internal consistency among the items.

**Table 1.** Percentage distribution of items used for measuring HCWs’ discriminatory attitudes towards PLHIV

Statement	% agreeing with the statements					Total (n=526)
	Doctor (n=160)	Nurse (n=135)	Medical technician (n=87)	Support staff (n=144)		
Those who have HIV and AIDS should not be allowed to mix freely with other people***	21.9	48.1	36.8	83.3	47.9
I prefer to refer persons with HIV and AIDS to other physicians/ care providers***	12.5	40.0	20.7	64.6	35.2
I do not want persons at high risk for HIV and AIDS as my patients***	12.5	40.0	20.7	64.6	35.2
Children with HIV and AIDS should not be allowed to attend public schools***	8.1	33.3	23.0	68.8	33.7
If I had a choice, I would not work with HIV/AIDS patients***	18.1	28.9	24.1	55.6	32.1
People who have HIV and AIDS should not be allowed to work***	6.9	28.1	23.0	66.0	31.2
Those who have HIV and AIDS should be forced to resign from their job***	19.4	29.6	28.7	55.6	30.4
I do not welcome those in my chamber, who are living with HIV and AIDS***	13.1	24.4	18.4	52.8	27.8
Needs of people with HIV should not be given priority***	12.5	30.4	24.1	36.8	25.7
I would not feel comfort if my other patients knew that I treated people with HIV and AIDS***	11.9	20.0	17.2	49.3	25.1
I would request my authority to remove me from the responsibility of caring PLIV***	6.9	24.4	12.6	44.4	22.6
I would not feel comfort if other physicians knew that I treated people with HIV and AIDS***	6.3	18.5	16.1	48.6	22.6
Medical students who are HIV-positive should not have the right to complete their degree***	9.4	16.3	13.8	40.3	20.3
Government should spend less money on HIV and AIDS, and more on other, more common diseases	18.1	24.4	13.8	20.8	19.8
Our country does not need any laws to protect people with HIV and AIDS from discrimination**	12.5	18.5	12.6	28.5	18.4
I do not have a responsibility to treat people with HIV and AIDS**	1.9	3.7	4.6	11.1	5.3

Chi-square value was significant (**p<0.01; ***p<0.001);

AIDS=Acquired immunodeficiency syndrome;

HCWs=Healthcare workers;

HIV=Human immunodeficiency virus;

PLHIV=People living with HIV

#### Knowledge on HIV and AIDS

A 10-item instrument was designed to measure the knowledge on HIV and AIDS [items can be seen in Hossain and Kippax (23)]. Items were selected based on the review of available literature (18, 19, 24–27), and the respondents were asked about the causes of HIV transmission, the means to prevent HIV, and how the disease progresses from HIV to AIDS. Responses were converted to correct and incorrect where ‘do not know’ was considered an incorrect response. For a correct response, a numerical value ‘1’ was allocated whereas for an incorrect response ‘0’ was allocated. Higher scores indicate greater knowledge on HIV and AIDS, and the reliability coefficient of these items was 0.71.

#### Irrational fear about HIV

Twelve items were selected to measure the irrational fear about transmission of HIV; the items can be seen in Hossain and Kippax (23). The items were adapted from Gerbert *et al*. (3), Herek *et al*. (28), and Herek and Capitanio (29). Responses to these items were converted into correct and incorrect where ‘do not know’ was considered an incorrect response. For a correct response, a numerical value ‘1’ was allocated whereas for an incorrect response ‘0’ was allocated. Higher scores indicate more irrational fear about HIV, and the reliability coefficient of these items was 0.91.

#### Other measures

In addition to the above-mentioned scale, the following variables were also considered in analyzing the correlates of discriminatory attitudes towards PLHIV: age, sex, education, region, religion, importance of religion in the HCW's life, marital status, occupation, having direct contact with HIV-positive people at work, treating HIV-positive people at the workplace, and the type of hospital where the HCWs are working.

### Statistical analysis

Data were analyzed at two levels. Correlation coefficients were used for examining the relationship between the dependent variable and other continuous and scale-independent variables in bivariate analysis, and one-way analysis of variance (ANOVA) was used for examining the association between the dependent variable and the categorical and ordinal-level independent variables in bivariate analysis. The variables that were found to be significant were entered into the multiple linear regression model to determine the correlates of discriminatory attitudes among the HCWs. The assumptions of linear regression, such as linearity, normality, etc., were checked.

The Bangladesh Medical Research Council and the Human Research Ethics Committee of the University of New South Wales, Sydney, Australia, approved the study.

## RESULTS

Sample characteristics of the participants of this study are presented in Table 2. The majority (62.5%) of the respondents were recruited from Dhaka. The large majority (78.9%) of the HCWs worked in the teaching hospitals. Almost sixty (59.9) percent of the respondents were male, and the average age of the respondents was 32 years. The average number of years of education of the respondents completed was 13.4, although it was much higher (18.3) among the doctors. About one-third (32.7%) of the respondents had had direct contact with HIV-positive people in their workplace.

**Table 2.** Sample characteristics of healthcare workers by their occupational position

Sample characteristics	% of healthcare workers				
	Doctor (n=160)	Nurse (N=135)	Medical technician (n=87)	Support staff (n=144)	Total (n=526)
Geographic area					
Dhaka	61.3	57.8	89.7	52.1	62.5
Chittagong	14.4	25.2	6.9	29.2	20.0
Sylhet	24.4	17.0	3.4	18.8	17.5
Type of hospital					
Teaching hospital	83.8	91.1	20.7	97.2	78.9
Non-teaching hospital	5.6	8.9	2.3	2.1	4.9
Diagnostic centre	10.6	-	77.0	0.7	16.2
Age (average in years)	32.2	27.7	33.8	34.7	32.0
Sex					
Male	63.1	11.1	94.3	81.3	59.9
Female	36.9	88.9	5.7	18.8	40.1
Marital status					
Married	52.5	47.5	67.8	72.2	59.1
Single and others	47.5	52.5	32.2	27.8	40.9
Years (average) of education	18.3	14.1	13.1	7.4	13.4
Religion					
Islam	86.9	63.7	95.4	82.6	81.2
Hindu, Christian, and Buddhist	13.1	36.3	4.6	17.4	18.8
Attended religious services in the last week					
Never	14.4	10.4	8.0	9.0	10.8
Once a week	18.1	8.1	19.5	23.6	17.3
2–3 days a week	15.6	28.1	25.3	29.2	24.1
Daily	51.9	53.3	47.1	38.2	47.7
Importance of religion in respondent's life					
Not too important	8.8	3.0	1.1	2.1	4.2
Somewhat important	24.4	4.4	4.6	1.4	9.7
Very important	66.9	92.6	94.3	96.5	86.1
Had any direct contact with HIV-positive person at work					
Yes	32.5	37.8	33.3	27.8	32.7
No	67.5	62.2	66.7	72.2	67.3

HIV=Human immunodeficiency virus

### Level and correlates of discriminatory attitudes

A number of statements were used for measuring the level of discriminatory attitudes in terms of both social- and healthcare-related issues (Table 1). A moderate level of discriminatory attitudes was observed among the HCWs who participated in this study: so, for example, 47.9% of the respondents mentioned that those who have HIV and AIDS should not be allowed to mix freely with other people. The level of discriminatory attitudes varied significantly across the different occupational roles: doctor, nurse, medical technician, and support staff.

It was observed that the level of discriminatory attitudes increased with age (r=0.086, p<0.05), importance of religion in their life (r=0.118, p<0.01), and irrational fear about transmission of HIV (r=0.583, p<0.001). On the other hand, discriminatory attitudes were the lowest among those with the highest schooling (r=−0.416, p<0.001) and accurate knowledge on transmission and prevention of HIV (r=−0.518, p<0.001).

Seven of nine categorical variables were significantly related to discriminatory attitudes (Table 3). These were: sex, religion, marital status, region where HCWs worked, type of hospital, occupation, and watching television. Bonferroni analysis of the difference in the level of discriminatory attitudes based on the type of hospitals showed that the level of discriminatory attitudes among the HCWs of teaching hospitals were significantly different from both non-teaching and diagnostic centres (p<0.001). However, the difference in the level of discriminatory attitudes between the HCWs of non-teaching and diagnostic centres was not statistically significant (p=0.659). Bonferroni analysis, in the context of occupation, showed that discriminatory attitudes of doctors were significantly lower than of nurses and support staff, and attitudes of support staff were more discriminatory compared to all other occupational categories.

**Table 3.** One-way ANOVA for discriminatory attitudes of healthcare workers with selected independent variables

Variable	No. of cases	Mean	95% CI	p value
Sex				0.046
Male	315	37.4	35.5–39.4	
Female	211	34.9	32.6–37.1	
Religion				0.010
Muslim	427	35.5	33.9–37.1	
Hindu, Christian, and Buddhist	99	40.4	36.7–44.1	
Marital status				0.022
Married	317	37.8	35.8–39.8	
Single	209	34.3	32.2–36.4	
Region of workplace				0.001
Dhaka	329	34.3	32.6–36.1	
Sylhet	92	36.4	32.9–39.9	
Chittagong	105	43.1	39.3–46.8	
Watching television				0.001
Yes	388	34.3	32.7–35.9	
No	138	42.4	39.3–45.5	
Type of hospital				0.001
Teaching hospital		38.6	36.9–40.3	
Non-teaching hospital	26	24.7	21.6–27.8	
Diagnostic centre	85	29.3	26.6–31.9	
Occupation				0.001
Doctor	160	28.8	27.2–30.4	
Nurse/paramedic	135	35.0	32.2–37.8	
Medical technician	87	31.4	28.4–34.4	
Support staff	144	49.2	46.1–52.3	
Have any direct contact with HIV-positive people				0.160
Yes	172	34.9	32.3–37.5	
No	354	37.2	35.4–38.9	
Have present in workplace while HIV-positive people were treated there				0.866
Yes	192	36.2	33.7–38.8	
No	334	36.5	34.7–38.3	

ANOVA=Analysis of variance;

CI=Confidence interval;

HIV=Human immunodeficiency virus

Multiple linear regression analysis was conducted to see the effect of each of the independent variables on discriminatory attitudes. However, multicolinearity was diagnosed before entering the independent variables into the multiple regression model. Multicolinearity was found between knowledge on transmission and prevention of HIV and irrational fear on transmission of HIV. The correlation coefficient of these two variables was −0.61 (level of irrational fear decreased with the increment of knowledge on transmission and prevention of HIV). Thus, knowledge on transmission and prevention of HIV was dropped from the regression analysis because of its lower level of correlation coefficient (r=−0.518) with the dependent variable compared to irrational fear about transmission of HIV (r=0.583). Multicolinearity was also found between age and marital status, occupational position, and education. Marital status was dropped from the regression analysis based on the collinearity diagnosis as age was working as the predictor of marital status: higher the age of the HCWs, lower the number of unmarried HCWs. Occupational position was dropped from the regression analysis as it is natural that doctors will have more years of education than others. It is the educational qualifications which determine who will take the position of doctor and who will take the position of support staff.

After adjusting for multicolinearity, the following variables, which were significant (p<0.05) in correlation and ANOVA, were entered into the multiple regression model: age, sex, years of schooling, watching television, religion, importance of religion in their life, region where the HCWs were working, type of hospital, and irrational fear about transmission of HIV. The results indicate that, being female, more years of education, less irrational fear about HIV, and working in non-teaching and diagnostic centres evoked less discriminatory attitudes (Table 4).

**Table 4.** Multiple regression model predicting discriminatory attitudes among healthcare workers

Variable	Unstandardized coefficients	Standardized coefficients	*t*	p value
	B	Beta		
Full model (R square=0.393, *F*_(10,515)_=33.365, p<0.0001)				
Sex (male as RC)	-3.068	-0.088	-2.398	0.017
Age (years in round figure)	0.047	0.027	0.712	0.477
Years of education completed	-0.393	-0.142	-3.286	0.001
Watching television (yes as RC)	0.538	0.014	0.365	0.715
Importance of religion	1.376	0.039	1.078	0.282
Region of workplace (Dhaka as RC)				
Chittagong	0.156	0.003	0.091	0.927
Sylhet	3.147	0.073	1.871	0.062
Type of hospital (teaching hospital as RC)				
Non-teaching	-7.949	-0.100	-2.695	0.007
Diagnostic	-6.080	-0.130	-3.300	0.001
Irrational fears about HIV	2.150	0.453	10.892	0.001
Final model (R square=0.385, *F*_(5,520)_=65.072, p<0.0001)				
Sex (male as RC)	-2.813	-0.080	-2.229	0.026
Years of education completed	-0.449	-0.162	-4.026	0.001
Type of hospital (teaching hospital as RC)				
Non-teaching	-8.273	-0.105	-2.998	0.003
Diagnostic	-6.540	-0.140	-3.818	0.001
Irrational fear about HIV	2.192	0.462	11.234	0.001

HIV=Human immunodeficiency virus;

RC=Reference category

## DISCUSSION

Discriminatory attitudes among the HCWs were very common in this study which reminds us of the importance of introducing appropriate intervention programmes to reduce stigma. The female HCWs had less discriminatory attitudes than the male HCWs. A quarter of the HCWs mentioned that they would not feel comfortable if their other patients and colleagues knew that they were involved in treating or providing care to HIV-positive patients. The discriminatory attitudes of the HCWs towards PLHIV were, thus, also associated with social and economic risks—influence of societal and familial prejudice and loss of earnings—as working with PLHIV is negatively viewed by the society (30).

Irrational fear about transmission of HIV strongly correlated with discriminatory attitudes at both bivariate and multivariate analyses. This finding is similar with the finding of Herek *et al*. who argued that fear produces discrimination towards PLHIV (28). Fear is associated with the positioning of HIV-positive people as ‘others’: homosexuals, sex workers, injecting drug-users, all of whom are already stigmatized in the society.

The type of hospital where the HCWs were working was a significant predictor of their discriminatory attitudes towards PLHIV. It was assumed that the HCWs who were working in the teaching hospitals would have less discriminatory attitudes than others. However, the findings of this study indicate that they had more discriminatory attitudes than others had. This may perhaps be explained by the differences in the type of HCWs interviewed from different hospitals. In the teaching hospitals, not all the HCWs were involved in providing care and treatment to PLHIV. Thus, the respondents interviewed from the teaching hospitals were from both the categories: who were involved in providing HIV treatment and who were not. On the other hand, the participating non-teaching and diagnostic centres of this study were mostly specialized in providing care, treatment, and diagnosis of sexually transmitted diseases and HIV. Thus, all the HCWs from the non-teaching and diagnostic centres who were interviewed were involved with either providing treatment for HIV or diagnosing HIV. Professional contact with HIV-positive people is likely to have reduced discriminatory attitudes as has been shown in other studies (1, 19, 20, 31, 32). However, in this study, professional contact with HIV-positive people had no significant effect on discriminatory attitudes.

The findings of this study have serious implications for public-health policy planners and human rights activists. High levels of discriminatory attitudes among the HCWs influence the decision-making process of the people living with HIV and AIDS and stop them from accessing voluntary counselling and testing, care, support, and treatment services (2, 9, 10, 18, 33–38). Additionally, experience of discrimination increases the depression and reduces the level of self-esteem among the HIV-positive people, which is adversely related to a number of issues, i.e. high-risk behaviour for transmitting HIV to others, low self-efficacy, and low adherence to antiretroviral therapy (2, 9, 10, 18, 33–38). For human rights activists, these findings are important because discrimination undermines the fundamental rights of HIV-positive people, including right to health, privacy, freedom from inhuman and degrading treatment or punishment, employment, and education.

This study is not, however, without limitations. First, self-reported discriminatory attitudes, instead of actual discriminatory behaviours, were studied. These attitudes were measured by some specific hypothetical questions, and hypothetical questions may suffer from bias due to the possibility of respondents providing responses that are socially acceptable rather than being correct which can be termed social desirability bias (16). There is also a limitation of genralizability of the findings of the study as the HCWs in the study were interviewed from three metropolitan areas only.

To have a full understanding of discriminatory attitudes of HCWs, they should be studied in the context of the broader socioeconomic milieu in which they live and work. First, class structure and power relations between the HCWs and the PLHIV should be considered. In Bangladesh, the general pattern of relationship between the HCWs and the patients is hierarchical with the HCWs positioned ‘top’ and patients positioned ‘bottom’. This positioning multiplies the degree of discrimination towards HIV-positive people. Second, the attitudes of HCWs are influenced by the society's existing perceptions towards HIV-positive people; for example, people will not visit those HCWs who provide treatment to HIV-positive people. Discriminatory attitudes among the general public constrain HCWs from treating HIV-positive people. Third, safety in the workplace is a concern for HCWs. The HCWs became more fearful in the absence of universal precaution in the healthcare system, and this also evokes discrimination towards PLHIV.

## ACKNOWLEDGEMENTS

The study received the UNFPA Research Fellowship Award from the Department of Population Sciences, University of Dhaka, Bangladesh, for conducting fieldwork. The authors thank the Department of Population Sciences for providing financial assistance.
